# Complex Interplay between the P-Glycoprotein Multidrug Efflux Pump and the Membrane: Its Role in Modulating Protein Function

**DOI:** 10.3389/fonc.2014.00041

**Published:** 2014-03-03

**Authors:** Frances Jane Sharom

**Affiliations:** ^1^Department of Molecular and Cellular Biology, University of Guelph, Guelph, ON, Canada

**Keywords:** P-glycoprotein (ABCB1), phospholipids, detergents, cholesterol, ATP hydrolysis, membrane partitioning, membrane transport, drug binding

## Abstract

Multidrug resistance in cancer is linked to expression of the P-glycoprotein multidrug transporter (Pgp, ABCB1), which exports many structurally diverse compounds from cells. Substrates first partition into the bilayer and then interact with a large flexible binding pocket within the transporter’s transmembrane regions. Pgp has been described as a hydrophobic vacuum cleaner or an outwardly directed drug/lipid flippase. Recent X-ray crystal structures have shed some light on the nature of the drug-binding pocket and suggested routes by which substrates can enter it from the membrane. Detergents have profound effects on Pgp function, and several appear to be substrates. Biochemical and biophysical studies *in vitro*, some using purified reconstituted protein, have explored the effects of the membrane environment. They have demonstrated that Pgp is involved in a complex relationship with its lipid environment, which modulates the behavior of its substrates, as well as various functions of the protein, including ATP hydrolysis, drug binding, and drug transport. Membrane lipid composition and fluidity, phospholipid headgroup and acyl chain length all influence Pgp function. Recent studies focusing on thermodynamics and kinetics have revealed some important principles governing Pgp–lipid and substrate–lipid interactions, and how these affect drug-binding and transport. In some cells, Pgp is associated with cholesterol-rich microdomains, which may modulate its functions. The relationship between Pgp and cholesterol remains an open question; however, it clearly affects several aspects of its function in addition to substrate–membrane partitioning. The action of Pgp modulators appears to depend on their membrane permeability, and membrane fluidizers and surfactants reverse drug resistance, likely via an indirect mechanism. A detailed understanding of how the membrane affects Pgp substrates and Pgp’s catalytic cycle may lead to new strategies to combat clinical drug resistance.

## Introduction

Many human cancers, including breast, kidney, and colon carcinomas, leukemias, multiple myeloma, and several pediatric cancers, develop multidrug resistance (MDR), which is a major obstacle to chemotherapeutic treatment (1, 2). MDR tumors are cross-resistant to a broad spectrum of structurally unrelated cytotoxic drugs, including the *Vinca* alkaloids (vinblastine, vincristine), anthracyclines (doxorubicin, daunorubicin), and taxanes. Physiologically, a complex network of ATP-binding cassette (ABC) proteins is involved in drug detoxification and protection of tissues from xenobiotics, including administered therapeutic drugs. Three of these ATP-driven drug efflux pumps are widely expressed in tumors, and have been linked to drug resistance; P-glycoprotein (Pgp; ABCB1), ABCG2 and ABCC1 (MRP1) (3, 4). Pgp and MRP1 exist as large single polypeptides, whereas ABCG2 is a “half-transporter” that functions as a homodimer. Together, these three proteins are able to carry out efflux of a wide range of anti-cancer drugs that are in common use clinically. The ability to inhibit the action of these drug efflux pumps has the potential to greatly improve the outcome of chemotherapy treatment, and the development of strategies to achieve this has been ongoing for many years.

Since Pgp was the first ABC drug efflux pump to be identified (5), and has been intensively studied for more than 35 years, it is the one we know most about. Hundreds of Pgp substrates have been identified over the years, including many clinically used drugs, chemotherapeutic agents, natural products, linear and cyclic peptides, amphiphiles, and fluorescent dyes (3, 4). The protein displays basal ATPase activity, which is often (but not always) stimulated by substrates, and it hydrolyzes ATP to power active transport, generating a drug concentration gradient across the membrane (6). Pgp and other ABC transporters are believed to operate by an alternating access model. The first step in transport involves binding of drug to the inward-facing conformation from the cytosolic side of the membrane. This is followed by a switch to the outward-facing conformation, which reorients the binding site to the extracellular side, resulting in drug release. ATP hydrolysis provides the energy for the switch between these two conformations. Despite recent progress in determining the high resolution structure of Pgp, there are still many gaps in our understanding of how the protein functions at the molecular level.

It was evident some time ago that the host membrane plays a central role in Pgp-mediated MDR (7, 8), and the complexity of this relationship has become more apparent as our knowledge of the protein has advanced. First, the membrane has a profound effect on Pgp’s lipophilic substrates, affecting both their lipid partitioning and transbilayer movement. Second, many aspects of Pgp structure (such as its stability and conformation) and function (including ATP binding, ATP hydrolysis, drug binding, and drug transport) are especially sensitive to the properties and composition of the surrounding membrane. It is important to understand how Pgp function is modulated by membrane properties, since this may influence transporter activity in tumors. Indeed, MDR was successfully reversed *in vitro* simply by altering the biophysical characteristics of the membrane (9). Changing membrane properties may thus be a useful approach for clinical reversal of MDR. The importance of the membrane in drug efflux also suggests additional approaches for development of new anti-cancer drugs and modulators with increased clinical effectiveness; for example, chemical modification of existing compounds may alter their interactions with the membrane, and their ability to access Pgp. If such strategies are to be successful, it is clearly important to have a detailed understanding of how the properties of the lipid environment affect all aspects of the Pgp catalytic cycle.

This review focuses on the complexity of the interactions of Pgp and its substrates with their membrane environment, the effect of these interactions on many different aspects of Pgp function, and how understanding them can help shed light on the molecular details of drug transport. Our current knowledge of Pgp has been gleaned from both *in vitro* and cellular studies of the transporter. The majority of these studies have been carried out with either rodent Pgp (Chinese hamster and mouse) or the human protein. The latter is clearly the clinically important homolog, but (as discussed further below) high resolution structures are currently available only for the mouse and *Caenorhabditis elegans* proteins. The rodent Pgps are highly active, relatively stable, and have proved extremely useful for *in vitro* studies of the structure and mechanism of action of the purified protein. The human protein, on the other hand, appears to be relatively unstable and is more difficult to express and purify [e.g., Ref. (10)]. The rodent Pgps are very closely related to the human homolog (e.g., 87% sequence identity and 93% sequence similarity between Chinese hamster and human Pgp), and only small differences in substrate specificity have been observed in cell-based transport assays. Recent work using purified hamster Pgp in a liposomal assay system to quantify drug interactions reported that results correlated very well with clinically relevant data on disposition of Pgp transport substrates in human subjects (11). Rodent Pgps thus remain very important models for understanding the function of the human protein.

## Pgp in Human Cancer: Its Role and Potential Modulation

P-glycoprotein expression is widespread in clinical cancer. The U.S. National Cancer Institute uses a panel of 60 tumor cell lines to identify and evaluate new anti-cancer agents. Expression of Pgp was detected in 39 of these cell lines, including renal and colon carcinomas, melanomas, and central nervous system tumors (12). Expression of Pgp was also highly correlated with resistance of these cell lines to anti-cancer drugs. Pgp has also been found in many human tumors (13, 14), with levels often increasing substantially after one or more rounds of chemotherapy, especially in acute myelogenous leukemia and lymphomas. Even very low levels of Pgp that are difficult to detect in tumor tissue significantly affect the sensitivity of cells to drugs. Although multiple mechanisms are undoubtedly responsible for drug resistance *in vivo* (15), Pgp is the single most important cause of MDR, representing an attractive target for intervention. Pgp expression has been linked to reduced responses to chemotherapy and poor clinical outcome for breast cancer, sarcomas, hematological malignancies such as leukemias, and pediatric cancers [reviewed in Refs. (1, 16)]. An important goal in cancer therapy has been the development of compounds that can effectively inhibit Pgp-mediated MDR. Such modulators (also known as chemosensitizers) have been identified based on their ability to reverse drug resistance in MDR cells *in vitro*. Pgp modulators commonly used in biochemical studies include verapamil and cyclosporin A. Since MDR is probably the single greatest barrier to successful chemotherapy, the ability to circumvent Pgp-mediated drug resistance could lead to improved treatment outcomes.

Three different strategies for defeating Pgp have been described (1); “engage” (co-administration of anti-cancer drugs and modulators), “evade” (the use of anti-cancer drugs that are poor Pgp substrates), and “exploit” (specific targeting of the Pgp molecule). Many clinical trials have been carried out to evaluate the hypothesis that co-administration of modulators would improve the effectiveness of chemotherapy drugs, thus leading to increased efficacy in patients [reviewed in Ref. (16)]. Early promising results obtained for pediatric cancers using cyclosporin A (17) generated optimism that this approach might be useful in tumors where Pgp expression is the primary cause of MDR. However, success in adult cancers has been elusive, in part because of poor clinical trial design and patient selection, and also because first and second generation modulators showed toxicity, sub-optimal effectiveness, and serious pharmacokinetic drug interactions (16, 18, 19). Since then, much more potent and less toxic third generation modulators have been developed to target Pgp specifically, such as LY336979 (zosuquidar) and XR9576 (tariquidar). However, they, too, failed to improve patient outcome in clinical trials [see Ref. (16)]. Several non-toxic plant natural products have also been proposed as MDR modulators, including curcumin (20), polyphenols (21), and flavonoids (22), however, these have not yet been tested in clinical trials. Given the disappointing outcome of the modulator strategy to date, it is not clear whether this line of attack will be successful. Designing novel approaches to specifically target Pgp in MDR will require detailed knowledge of the various steps in the catalytic cycle.

## Vacuum Cleaner and Flippase Models for Pgp Function

### Pgp as a hydrophobic vacuum cleaner

**Figure 1:**
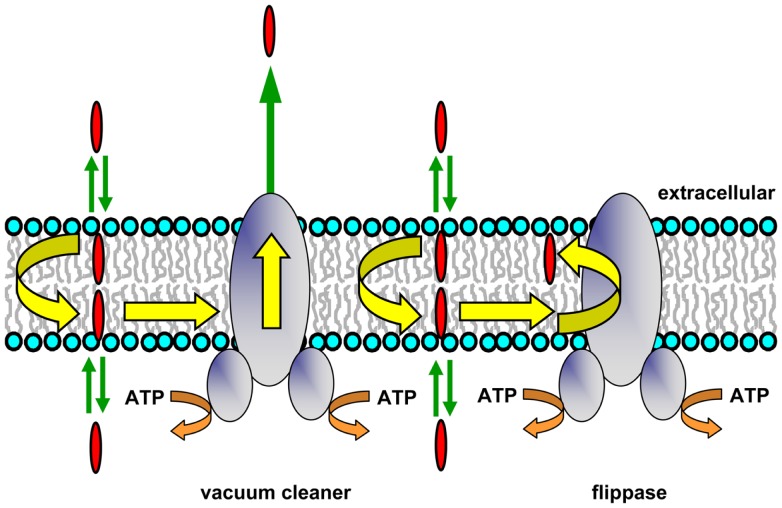
**Hydrophobic vacuum cleaner and flippase models of Pgp function**. In the vacuum cleaner model, drugs partition into the membrane, spontaneously translocate to the cytoplasmic leaflet, and gain access to the Pgp substrate-binding pocket from within the bilayer interior. They are subsequently effluxed into the extracellular aqueous phase. In the flippase model, drugs partition into the membrane, spontaneously translocate to the inner leaflet, interact with the Pgp substrate-binding pocket, and are then flipped to the outer membrane leaflet. The drug concentration will be higher in the outer leaflet compared to the inner leaflet, and a concentration gradient is generated when drugs rapidly partition from the two membrane leaflets into the aqueous phase on either side of the membrane.

The compounds that Pgp transports are typically lipophilic, so they accumulate within the lipid bilayer. Pgp substrates are also generally amphipathic molecules, and rather than distributing uniformly in the hydrophobic core of the lipid bilayer, they align themselves in the interfacial region. NMR studies have shown that they tend to concentrate between the lipid headgroup and the first few carbon atoms of the lipid acyl chains (23). Early work strongly suggested that Pgp effluxes drugs directly from the membrane, rather than the aqueous phase (6). Acetoxymethyl ester derivatives of various fluorescent calcium and pH indicators entering intact cells from the extracellular side are intercepted and extruded without entering the cytosol (24). A Förster resonance energy transfer (FRET) study in which the transporter was photolabeled with the lipophilic probe iodonaphthalene-1-azide showed that the substrate doxorubicin was present within the membrane close to Pgp (25). This suggested that Pgp may interact with its substrates within the membrane and subsequently efflux them to the extracellular medium. These observations and others led to the proposal by Higgins and Gottesman that the transporter was a hydrophobic “vacuum cleaner” (Figure 1) responsible for removal of potentially harmful lipophilic compounds from the membrane (26). Later work showed that the rate of Hoechst 33342 efflux by Pgp was directly proportional to the membrane concentration of the fluorescent dye, but inversely proportional to its aqueous concentration, supporting the idea that transport takes place from the bilayer interior (27). In addition, studies employing a deletion mutant of Pgp showed that the transmembrane (TM) domains of the transporter were sufficient to bind drug substrates (28). The vacuum cleaner model is now widely accepted, and was a key concept in our understanding of the relationship between Pgp and its membrane environment.

Transport studies using reconstituted Pgp, plasma membrane vesicles, and intact MDR cells showed that several fluorescent substrates (Hoechst 33342, LDS-751, and a rhodamine derivative) were extracted from the cytoplasmic membrane leaflet (29–31). FRET studies designed to pinpoint the location of the binding sites for Hoechst 33342 and LDS-751 also showed that they were situated within the TM regions of Pgp, in the cytoplasmic leaflet of the membrane (32, 33). The recent X-ray crystal structures of Pgp from mouse (34) and *C. elegans* (35) have allowed a closer view of the potential routes that substrate molecules may follow from the bilayer inner leaflet into the binding pocket of the protein (see below).

### Pgp as a drug and lipid flippase

P-glycoprotein may be envisaged as expelling its substrates directly into the extracellular medium, which would have an energy cost for lipophilic species. The protein has also been proposed to operate as a drug translocase or flippase (Figure 1), moving its substrates from the inner to the outer leaflet of the membrane (26). This mechanism requires that drug molecules have a specific localization within each bilayer leaflet, rather than being randomly distributed in the hydrophobic core. An NMR study of Pgp substrates and modulators confirmed that this is the case, and showed that these molecules align with the phospholipid acyl chains (23). After reaching the outer leaflet, substrates would then either passively diffuse into the extracellular aqueous phase (a very fast process), or move back to the inner leaflet by spontaneous flip–flop. In order to maintain a substrate concentration gradient across the membrane, the rate of passive transbilayer flip–flop of substrate would need to be slower than the rate of Pgp-mediated flipping, so that its concentration remains higher in the outer leaflet. Indeed, the rate of drug movement between leaflets is variable, and can be quite slow, with half-times ranging from minutes to hours depending on the structure of the compound (36, 37). The flippase model involves delivery of drug to the outer leaflet, followed by rapid partitioning into the extracellular medium, while in the vacuum cleaner model, drug is delivered to the extracellular medium, followed by rapid partitioning into the outer leaflet. Since the same equilibrium state is reached in each case, it is not currently possible to distinguish experimentally between these two models, and they are not mutually exclusive. Energetic considerations suggest that release of a dehydrated lipophilic substrate into the outer membrane leaflet would be energetically more favorable than transfer into the aqueous phase followed by hydration, but it is possible that both of these locations are accessible to the substrate-binding pocket of Pgp during the transport process.

There is substantial evidence supporting the flippase model for Pgp function. Indeed, the protein is able to act as an outwardly directed flippase for several fluorescent phospholipid and glycosphingolipid molecules in both intact cells and reconstituted proteoliposomes. Cells overexpressing native or recombinant Pgp showed altered distribution of fluorescent phosphatidylcholine (PC), phosphatidylethanolamine (PE), and sphingomyelin (SM), accumulated lower amounts of both short- and long-chain fluorescent phospholipid derivatives, and displayed increased outward transport of these analogs, which decreased after treatment with Pgp modulators (38–42). These observations strongly suggested that the lipids were Pgp substrates. Sharom and co-workers showed directly that purified Pgp reconstituted into proteoliposomes can act as a broad-specificity, outwardly directed flippase for a variety of both short-chain and long-chain nitrobenzo-2-oxa-1,3-diazole (NBD)-labeled phospholipids (43). Phospholipids with the fluorescent NBD group on both the acyl chain and the headgroup were translocated. Simple glycosphingolipids, such as glucosyl- and galactosylceramide were also flipped by reconstituted Pgp, as was lactosylceramide, although at a greatly reduced rate (44). Species with headgroups larger than two sugar residues are unlikely to be substrates. Like drug transport, phospholipid and glycolipid flippase activity required ATP hydrolysis, and was inhibited by the phosphate analog, *ortho*-vanadate. Flippase activity was inhibited in a concentration-dependent manner by known Pgp substrates, and inhibitory potency was highly correlated with their Pgp binding affinity, suggesting that drugs and membrane lipids follow the same route through the transporter. Taken together, these observations suggest that Pgp-mediated drug efflux probably takes place by a flippase-like mechanism.

### Interaction of lipids and lipid-like molecules with Pgp

**Figure 2:**
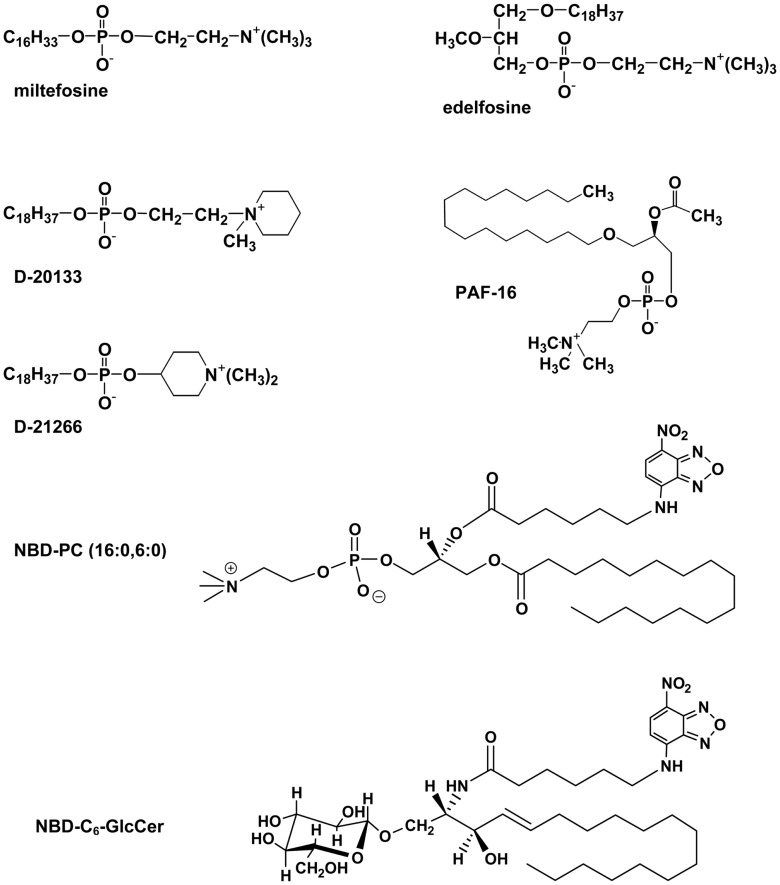
**Structures of some lipid-based Pgp substrates**.

Eckford and Sharom (45) used several functional screens to identify a number of lipid-based molecules that interact directly with Pgp with high affinity (Figure 2). These compounds resulted in inhibition of ATPase activity, competition for transport of two drugs, and competition for translocation of NBD–PC (16:0, 6:0), suggesting that they are substrates for Pgp. Included in these lipid species are platelet-activating factors (PAFs), ether–phospholipid signaling molecules that are probably endogenous substrates for the transporter (46, 47). Pgp is also able to bind and transport hexadecylphosphocholine (also known as miltefosine), an anti-cancer drug structurally related to PC (48). Other anti-cancer agents such as the alkyl phospholipids, edelfosine, and ilmofosine, and the related compounds, D-20133 and D-21266, also interact with the transporter, leading to resistance (45, 48). In support of these results, yeast cells expressing Pgp were previously reported to be resistant to the cytotoxicity of edelfosine (49). Pgp–lipid interactions thus have important consequences for cancer treatment using these lipid-based drugs.

Metabolic labeling of Pgp, followed by purification and organic solvent extraction, was used to isolate and identify the membrane lipids closely associated with Pgp (50). These proved to be enriched in PE and to a lesser extent phosphatidylserine (PS), but contained little PC or SM, despite the fact that these choline phospholipids are major components of the plasma membrane from which the transporter was solubilized. Thus Pgp associates preferentially with certain phospholipids; in this respect, it is interesting that PE and PS are enriched in the inner leaflet of the plasma membrane, whereas PC and SM are found primarily in the outer leaflet.

A recent mass spectrometry (MS) study (51) examined lipid interactions with Pgp using purified mouse protein in micelles of the detergent *n*-dodecyl-β-d-maltoside (DDM). Gas phase Pgp ions were released and retained only one to two detergent molecules, possibly in the binding pocket. MS could detect simultaneous binding of lipids, drugs, and nucleotides. Estimates of apparent *K*_d_ values, which were in the micromolar range, indicated that negatively charged lipids bind to Pgp more favorably than zwitterionic lipids, and both steric effects and headgroup charge modulated binding. Binding of the cyclic peptide, cyclosporin A, was observed to promote subsequent lipid binding. Interestingly, cardiolipin, a bulky anionic mitochondrial lipid, was also able to bind to Pgp. It was suggested that lipid molecules could interact with the transporter inside the large binding cavity and also at the interface between the protein and the lipid bilayer. This novel approach shows promise in the direct study of drug and lipid interactions with Pgp.

### Physiological role of Pgp–lipid flippase activity

The physiological significance of Pgp’s lipid flippase activity is still the subject of speculation. The protein may be the primary means of exporting PAFs *in vivo*, by translocating them from the cytoplasmic to the extracellular membrane leaflet, from which they can diffuse into the aqueous external environment. Other probable endogenous Pgp substrates include steroid hormones such as aldosterone (52) and β-estradiol-17β-d-glucuronide (53). Indeed, the transporter is known to interact with several steroids (54, 55), and a role in transporting these hormones could explain Pgp expression in the adrenal gland. Pgp may also play a physiological role in the biosynthesis of complex glycosphingolipids in the Golgi by translocating glucosylceramide from the cytoplasmic to the luminal membrane leaflet. The protein most closely related to Pgp, ABCB4 (78% sequence similarity), acts primarily as a PC-specific flippase in the liver, exporting phospholipid into the bile (56, 57), although it can also transport some drugs at a low rate (58). Thus Pgp and ABCB4 may function in a similar manner.

It seems unlikely that the primary role of Pgp *in vivo* is that of a lipid flippase, because the rate of flipping is relatively low (43, 44), and Pgp could not rescue a knockout of ABCB4, even when both proteins were expressed in the liver canalicular membrane (59). However, differing expression levels of each protein could confound this observation. Another consideration is the existence of the plasma membrane aminophospholipid translocase, ATP8a1, which is a member of the P4-ATPase family. This protein normally translocates PE and PS to the inner leaflet, thus maintaining bilayer asymmetry (60). If Pgp moves these endogenous lipids from the inner to the outer leaflet at a significant rate, it would counteract the action of the translocase, resulting in a futile cycle of phospholipid flip–flop accompanied by wasteful hydrolysis of ATP.

Other mammalian ABC superfamily members are known to operate as physiological lipid transporters or flippases (61–63). Three proteins in the ABCA subfamily (ABCA1, ABCA7, and ABCA4) were shown to translocate fluorescent phospholipids in reconstituted systems (64). ABCA1 and ABCA7 transported lipids from the cytoplasmic to the extracellular leaflet, whereas ABCA4 is the only known eukaryotic ABC protein to move substrate in the opposite direction.

## Pgp Structure

**Figure 3:**
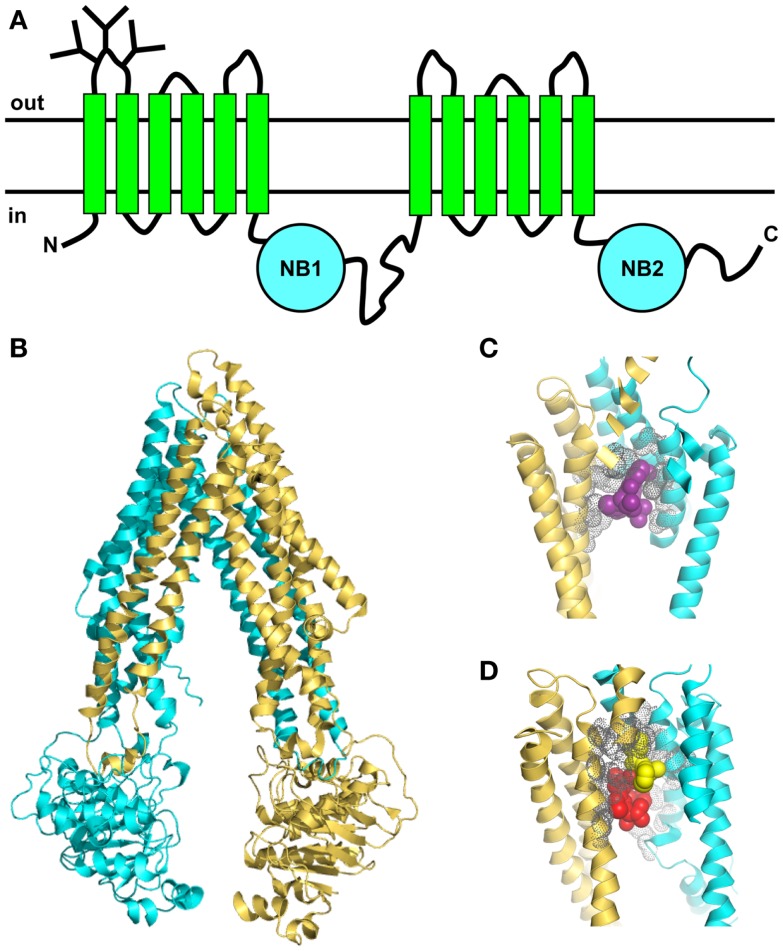
**Topology and X-ray crystal structures of Pgp**. **(A)** The membrane topology shows two homologous halves, each with six TM segments and a NB domain on the cytoplasmic side, which binds and hydrolyzes ATP. **(B)** X-ray crystal structure of mouse Pgp (34) shows an inward-facing conformation in the absence of nucleotide (PDB 3G61). **(C,D)** The drug-binding pocket of mouse Pgp as seen in the X-ray crystal structure. **(C)** Close-up view (4.4 resolution) of one molecule of the cyclic peptide modulator QZ59-RRR (magenta, space-filling format) occupying the middle site inside the drug-binding pocket, with the volumes of nearby side-chains shown in gray (PDB 3G60). **(D)** Close-up view of two molecules of the cyclic peptide modulator QZ59-SSS occupying the upper and lower sites (yellow and red, respectively, space-filling format) inside the drug-binding pocket, with the volumes of nearby side-chains shown in gray (PDB 3G61).

Like other ABC proteins, Pgp comprises two homologous halves, each consisting of six TM segments, and two nucleotide-binding (NB) domains on the cytosolic side where ATP binds and is hydrolyzed (Figure 3A) (65, 66). The NB domains of ABC proteins contain three highly conserved sequences; the Walker A and B motifs (found in many ATP-binding proteins) and the C (or ABC signature) motif that is unique to this superfamily (67). It is now clear that nucleotide binding to ABC transporters is driven by dimerization of the NB domains (66, 68), which is essential for ATP-driven transport. The X-ray crystal structures of several isolated NB subunits of bacterial ABC proteins showed an interdigitated head-to-tail arrangement, a so-called sandwich dimer. Two ATP molecules are bound at the interface of the sandwich dimer, each interacting with the Walker A and B motifs of one NB domain and the C motif of the partner domain. These stable symmetric dimers are only observed when the NB domains are in an inactive state, either by mutation of an amino acid residue essential for catalysis, or in the absence of Mg^2+^ (69, 70). Thus, they probably do not represent a true catalytic intermediate.

Early electron microscopy (EM) studies provided the first glimpses of Pgp’s three-dimensional structure [reviewed in Ref. (71)]. A medium-resolution EM structure of Chinese hamster Pgp at 8-Å resolution was obtained by cryo-electron crystallography of two-dimensional crystals (72). The protein contained bound nucleotide (AMP-PNP), and showed an asymmetric conformation. Other EM studies showed that the two NB domains of Pgp are closely apposed, leading to a closed conformation that was attributed to a sandwich dimer (73, 74).

In the past few years, important advances have been made in understanding the nature of the Pgp-drug-binding pocket [e.g., Ref. (32, 33, 75, 76)]. Mutagenesis studies suggested that the pocket was located at the interface between the two TM halves (77), and this was later confirmed by photoaffinity labeling in conjunction with MS (78, 79). The first X-ray crystal structures of mouse Pgp in an inward-facing conformation appeared in 2009 (34). The structures were determined in the absence of nucleotide, with and without bound cyclic peptide substrates, which are located in a drug-binding pocket within the TM regions of the protein. This large flexible cavity is made up of two bundles of six helices, each composed of portions from both the N- and C-terminal halves (Figure 3B). This phenomenon, known as domain swapping, has also been reported for other ABC exporters. The pocket is accessible from the cytoplasmic leaflet of the membrane, as expected. The drug-binding region is partially lined with aromatic and hydrophobic residues, which are proposed to bind substrates via hydrophobic and van der Waals interactions. Different stereoisomers of the same cyclic peptide substrate are held in different sub-sites by a unique set of interactions, and two molecules of the same substrate can fit into the pocket simultaneously, via different sets of interactions (Figures 3C,D). The presence of overlapping sub-sites inside the binding pocket helps to explain the extraordinary range of chemical structures that interact with Pgp. Although the mouse structures are not of very high resolution, they provided some useful information that will help to guide future studies. However, they have not settled any of the outstanding controversies in the field, such as the extent of the separation and movement of the NB domains during the catalytic cycle. Some new mouse Pgp structures show wider separations of the two NB domains (80). One structure shows a complex with a nanobody inhibitor, which appears to inhibit ATP hydrolysis by binding to an epitope on the N-terminal NB domain, thus preventing dimerization of the two domains.

A higher resolution structure (3.4 Δ) of *C. elegans* Pgp in an inward-facing conformation, without bound substrate or nucleotide, appeared in 2012 (35). It shows a portal leading from the cytoplasmic side of the bilayer to the central cavity where substrates are presumed to bind. This structure is compatible with decades of biochemical analysis on the human protein, and helps to explain perplexing functional data on the Phe335Ala mutant. Homology models of human Pgp derived from the *C. elegans* and mouse Pgp structures differ significantly in their orientation of TM helices 3–5, and recent work suggests that the *C. elegans* model is most likely correct (81).

Recent simulation studies of mouse Pgp in a model membrane showed a wide range of NB domain separations, underscoring the apparent high flexibility of the inward-facing protein conformation (82). Of great interest is the observation that the acyl chain of a membrane lipid slid into the cleft between TM helix 4 and TM helix 6, and remained there for the duration of the 50-ns simulation. The tip of the penetrating chain, which was more than 10 carbon atoms long, made contact with several amino acid residues lining the substrate-binding pocket that are known to be involved in drug binding. It is possible that the binding pocket of Pgp may be occupied by endogenous membrane phospholipids in the absence of drug substrates, in line with the idea that it also functions as a low activity lipid flippase. Occupation of the binding pocket by lipids might also explain the high basal ATPase activity observed for Pgp from mouse and Chinese hamster. If these lipids bind with relatively low affinity, they may be displaced by high affinity drug substrates, which would thus be transported preferentially.

To date, only inward-facing conformations of Pgp have been obtained using crystallographic studies, and there is still no nucleotide-bound structure available. Until these two “missing links” are obtained, it will be difficult to make progress on understanding the structural changes that take place during the catalytic and transport cycles. The difficulty in crystallizing Pgp in the outward-facing or nucleotide-bound forms might arise from either the high dynamic flexibility or low stability of these conformations.

## Model Systems for Studying the Interplay between Pgp and Lipids

The vacuum cleaner and flippase models predict that Pgp will be very sensitive to its membrane microenvironment. Because of the complexities of intact cells, they have limited usefulness in exploring the relationship between transporter function and membrane properties. Plasma membrane fluidity can be altered by growing cells in medium containing saturated fatty acids, which alters the endogenous lipid composition of cellular membranes. Membrane properties can also be changed by adding exogenous amphiphiles, or membrane rigidifying or fluidizing agents [e.g., Ref. (9)]. Experimental observables are typically restricted to indirect measures of Pgp function, such as total cellular drug uptake, or cell survival in the presence of drug.

Simpler model systems have provided unique opportunities to explore the structure and function of Pgp. Mammalian MDR cell lines selected with cytotoxic drugs can express very high levels of Pgp [up to 30% of the total plasma membrane protein (83)], and have been popular choices as the starting material for model systems. Sealed inside-out plasma membrane vesicles have been used for studies of ATP-driven drug transport [see Ref. (6)], and also as a source of purified active protein for biochemical and spectroscopic characterization. These studies have typically relied on the use of exogenous agents to alter membrane fluidity. For example, the ability of Pgp in canalicular membrane vesicles to transport daunorubicin and vinblastine was reduced two- to four-fold by benzyl alcohol, a known membrane fluidizer (84).

Several research groups have reported successful purification of Chinese hamster, mouse, and human Pgp from drug-selected MDR cell lines, or cells expressing recombinant protein [reviewed in Ref. (6)]. The use of solubilizing detergents is clearly essential in the extraction and purification of Pgp from native cell membranes. The choice of detergent is a critical step, and is typically governed in the first instance by the necessity of maintaining various functions of the protein, including ATP binding, ATP hydrolysis, drug binding, and drug transport. A second consideration is the compatibility of the chosen detergent with reconstitution, if this is a desired goal (85). Detergents with very low critical micelle concentrations (CMCs), such as Triton X-100, are usually very difficult to remove by dialysis, gel filtration chromatography, or dilution, and are not a good choice for reconstitution. The mild detergents 3-[3-(cholamidopropyl) dimethyl amino]-1-propanesulfonate (CHAPS) and octyl-β-d-glucoside (OG) have relatively high CMC values, and have been used successfully for purification and functional reconstitution of both Chinese hamster and human Pgp from mammalian cells (86–89).

Purified Pgp of mouse or Chinese hamster origin typically displays high levels of both basal and drug-stimulated ATPase activity (2–3 μmol/min/mg), and can carry out ATP-dependent active transport of drugs and hydrophobic peptides after reconstitution [summarized in Ref. (90)]. However, the basal rate of ATP hydrolysis of purified human Pgp is much lower than that of the rodent proteins, and it appears to require the presence of both drug substrates and lipids for activity. The rodent and human proteins are highly homologous, and the underlying reason for this difference in behavior is not clear. Human Pgp was recently reconstituted into lipid nanodiscs composed of *Escherichia coli* lipids, where it retained robust levels of both basal and drug-stimulated ATPase activity (91). Surface plasmon resonance was used to probe conformational changes in Pgp associated with progression through the ATP hydrolysis cycle. This novel nanodisc platform may prove useful in the further study of human Pgp.

Many of the purified Pgp preparations described in the literature employ the addition of exogenous lipids to maintain its function. Very few research groups have isolated Pgp in the absence of added exogenous lipids (50, 92). This approach allowed the development of experimental protocols to reconstitute Pgp into proteoliposomes of defined phospholipids, either synthetic or natural mixtures (92–94). Using these model systems has permitted detailed investigation of how Pgp function is modulated by membrane properties, leading to unique insights into transporter behavior. Further details of these studies are provided below.

## Detergent Interactions and Effects on Pgp Function

The interaction of Pgp with detergents has been examined from two different aspects. First, the effectiveness of different detergents in solubilization and functional reconstitution of Pgp has been studied by several groups. Coupled with this has been the investigation of how exposure to various detergents stabilizes or destabilizes the protein. Second, the role of detergents as specific Pgp substrates and modulators has been explored, with a view to probing the nature of their interactions with the drug-binding pocket.

Sharom and co-workers established a protocol for partial purification of Chinese hamster Pgp with high levels of ATPase activity using the zwitterionic detergent CHAPS (95), in the absence of any exogenously added lipids. This allowed subsequent exploration of the effects of various detergents on the protein’s activity and stability (96). Only the high CMC detergents, CHAPS and OG, were able to preserve ATPase activity at higher concentrations (4 mM for OG, 10 mM for CHAPS), whereas Triton X-100, digitonin and SDS resulted in complete loss of activity at 100 μM levels. The ability of CHAPS to preserve Pgp ATPase activity, while other detergents caused loss of activity was later confirmed by Orlowski et al. (97). They noted that drug stimulation of ATPase activity was restored after dilution of detergent to a concentration below its CMC. Even after almost complete removal of CHAPS from purified Pgp by dialysis, 80–90% of the ATPase activity remained (96), suggesting that the protein still retained a substantial annual lipid layer around it, which provided protection from denaturation. Later work used metabolic labeling to show that highly purified Pgp in CHAPS solution retained 53–56 tightly bound phospholipids per protein molecule (50). This amount may be sufficient to surround the TM domains of the protein, thus assisting in maintaining its native conformation and function.

The non-ionic detergents DDM and zwittergent 3–12 were reported to inactivate Chinese hamster Pgp function at low concentrations, below their CMCs, while OG inhibited activity in the millimolar range (98). In all cases, loss of ATPase activity and reduced drug-binding activity were prevented by the addition of 0.2% w/w of a crude lipid mixture. It was suggested that these detergents disrupted the lipid–protein interface, which is essential for Pgp to maintain its functional conformation. Similarly, the non-ionic *n*-alkyl-β-d-maltosides and *n*-ethyleneglycol monododecyl ethers were reported to reduce Pgp ATPase activity at concentrations well below their CMC values (99).

Naito and Tsuruo carried out the purification of human Pgp from MDR K562 cells using a panel of 12 detergents, and assessed its function following reconstitution into proteoliposomes (100). They reported that only cholate, glycocholate, and taurocholate were able to extract the protein and maintain its activity. However, the measured ATPase and transport activities were extremely low, likely as a result of protein denaturation during immunoaffinity elution. The overall conclusion from all of these studies is that the sensitivity of Pgp to inactivation by detergents depends on the lipids present in its immediate environment.

P-glycoprotein was reported to be dimeric or multimeric in the plasma membrane of some MDR cells (101–103), however, the minimal functional unit of the protein appears to be a monomer (104). Poruchynsky and Ling reported that detergent extracts from hamster and human MDR cell lines contained Pgp in an oligomerized form (105). CHAPS resulted in a high proportion of oligomerized Pgp, whereas only monomers were found after SDS treatment, suggesting that the nature of the detergent is important in either the formation or preservation of oligomers. Both Pgp monomers and oligomers appeared functional, in that they bound photoactive nucleotide and drug analogs. However, other studies have failed to find any evidence of Pgp dimerization using both biochemical and genetic approaches (106). The existence and functional role, if any, of oligomeric forms of Pgp is, therefore, still not clear.

Doige and Sharom first noted that Triton X-100 stimulated Pgp ATPase activity at low concentrations and inhibited it at higher concentrations, suggesting that it interacted specifically with the transporter (96). Triton X-100 was also observed to inhibit photoaffinity labeling of Pgp by the substrate ^3^H-azidopine at low concentrations (107, 108). It was later confirmed to be a high affinity substrate for Pgp (*K*_d_ = ~0.4 μM) using fluorescence quenching of purified protein (109). Several cationic amphiphiles, including the detergents benzalkonium chloride, methylbenzethonium chloride, cetylpyridinium chloride, and dodecyltrimethylammonium chloride, also appear to be Pgp substrates (107, 108). Both drug-selected MDR cells and Pgp transfectants showed cross-resistance to these compounds, which was effectively sensitized by the modulator verapamil. The synthetic surfactant, nonylphenol ethoxylate, which is a common component of various household detergents, was identified as a constituent of human urine, and *in vitro* studies confirmed that it is also a Pgp substrate (110). These findings indicate that the compound is excreted into the urine *in vivo* by kidney Pgp, in keeping with the proposed physiological role of the transporter in elimination of potentially toxic compounds.

Seelig’s group further pursued the idea that detergents may be Pgp substrates using the non-ionic species Triton X-100, C_12_EO_8_ (dodecyloctaglycol), and Tween 80 (polysorbate-80) (111). The bell-shaped curves obtained for the effect of these detergents on Pgp ATPase activity suggested that they behaved like substrates, and interacted directly with Pgp with high affinity (*K*_d_ values ~1 μM). Thermodynamic measurements showed that the ethoxyl groups of these detergents formed H-bonds with donor amino acid residues in the TM regions of Pgp, and their binding affinity increased with the number of these groups. Further investigation used a large series of polyoxyethylene alkylether detergents with varying numbers of methylene and ethoxyl residues (C_m_EO_n_), to systematically dissect the role of hydrophobic and H-bonding in binding of these compounds (112). Thermodynamic parameters indicated that detergent binding to the Pgp substrate cavity is driven exclusively by H-bonding or weak electrostatic forces, and not by hydrophobic interactions. Binding to Pgp is only achieved if the detergent has at least two hydrogen bond acceptor units within its chemical structure, supporting earlier work that used a large number of structurally diverse substrates (113). It is energetically unfavorable for the non-polar moiety of the detergent (the methylene chain) to enter the substrate-binding pocket, and it likely remains in contact with the hydrophobic lipid bilayer, where it may assist in flipping of the molecule to the other leaflet (112). This concept appears counter-intuitive, since the existence of hydrophobic and aromatic interactions between bound substrates and the surrounding cavity was suggested based on the X-ray crystal structures of mouse Pgp (34), and many QSAR studies have also stressed the importance of non-polar groups in binding to the transporter. However, the primary role of these groups may actually be to promote partitioning of the substrate into the bilayer, where it can then gain access to the protein’s binding cavity.

Some detergents were reported to produce unexpectedly large down-modulation of Pgp ATPase activity, which was not a result of membrane disordering (114). The ratio of the free energy of detergent partitioning into the membrane (from *K*_lip_) and the free energy of detergent binding to Pgp from within the membrane (from *K*_dlip_) appeared to control the rate of ATP hydrolysis induced by the detergent, and probably the rate of its transport. Deviations from an optimal ratio of these free energies led to reduced rates of ATP hydrolysis and, by extension, transport. It was suggested that similar principles apply to drugs that are Pgp substrates and modulators.

## Perturbation of Lipid Bilayers by Pgp

Early work on drug-selected MDR cell lines indicated that overexpression of Pgp led to secondary changes in the properties of their plasma membrane [summarized in Refs. (107, 115)]. It seemed likely that insertion of this large hydrophobic protein perturbs the physiochemical properties of the membrane, especially in cell lines where Pgp may represent as much as 20–30% of the total plasma membrane protein (83, 92). Loe and Sharom used the fluorescent probe merocyanine 540 to examine the local microenvironment in the plasma membrane of various CHO cell lines expressing Pgp (107). This negatively charged dye localizes in the outer leaflet of the plasma membrane, and the extent of its partitioning into membranes depending on the lipid packing density. Increasing levels of cellular drug resistance led to a progressive shift in the mean cell fluorescence to lower levels, indicating that the molecular packing of lipids in the outer leaflet of MDR cells increases with higher levels of Pgp expression.

The availability of purified Pgp led to further studies in reconstituted systems. Differential scanning calorimetry was first carried out by Romsicki and Sharom, using the synthetic phospholipid dimyristoyl-PC (DMPC) as the host lipid (116). Inclusion of increasing mole ratios of Pgp led to a decrease in the melting temperature of the bilayer, *T*_m_. The cooperativity of the lipid melting transition was greatly reduced, and the transition enthalpy, ΔH, decreased linearly as the Pgp content increased to a lipid:protein ratio of 16:1 (w/w). Pgp was found to perturb a large number of bilayer phospholipids, preventing 375–485 of them from participating in the phase transition. As the Pgp content of the bilayers increased, the transition enthalpy was observed to increase again, an effect likely arising from either aggregation/oligomerization of the transporter, or a change in its mode of interaction with the bilayer. Oleinikov et al. explored the perturbing effects of Pgp in its native environment by forming monolayers from membrane fractions derived from a series of MDR cell lines in which the Pgp content varied from 0 to 32% (w/w) (117). Compression of the Langmuir–Blodgett monolayers showed that 11 and 24% Pgp decreased monolayer stability without altering the surface area, while 32% Pgp increased both the stability and the surface area. Raman spectroscopy indicated that the presence of 32% Pgp reduced the lipid transition temperature by 7°C, and the number of perturbed lipids per protein was estimated at 400–500 (117), thus confirming the results seen in reconstituted systems (116).

Callaghan and co-workers reported that incorporation of Pgp into bilayers of PC–PE altered their overall fluidity, increased their permeability to polar compounds, and modified the packing organization of fluorescent lipid probes (115). The presence of the transporter also increased the flip–flop rate of short-chain phospholipids between bilayer leaflets, but this did not require ATP hydrolysis, so it likely takes place by an indirect mechanism.

Similar types of perturbing effects have previously been noted when other large hydrophobic proteins are inserted into lipid bilayers [e.g., Refs. (118, 119)]. Effects specific to Pgp may arise from its oligomerization behavior, and the ability of the transporter to interact with, and translocate, membrane lipids.

## Modulation of Pgp Functions by the Membrane

The availability of purified Pgp, both in detergent solution and reconstituted proteoliposomes, coupled with the development of biochemical and spectroscopic techniques for assessing many aspects of Pgp function, have greatly increased our understanding of the ways in which the lipid environment can modulate the transporter. The catalytic cycle of Pgp involves several steps, including nucleotide binding, drug binding from within the membrane, nucleotide hydrolysis, and drug translocation. Each of these steps may be subject to the influence of the surrounding membrane. In recent years, greater emphasis has been placed on the importance of measuring thermodynamic and kinetic constants for the various steps, since these provide a sound basis for construction of a detailed, experimentally testable model of the catalytic cycle.

### Drug-membrane partitioning

P-glycoprotein substrates are typically lipid-soluble and amphipathic, and it is now clear that their binding to the protein takes place in two steps: partitioning into the lipid bilayer, followed by interaction with the substrate-binding pocket located within the TM domains. The presence of a drug within the membrane inner leaflet is thus a primary determinant of its recognition by the transporter. Amphipathic molecules are not randomly distributed in the bilayer, but orient themselves in an anisotropic manner at the lipid–water interface, parallel to the long axis of the membrane phospholipids (23, 120, 121). Their polar moieties interact with water and phospholipid headgroups, and (depending on the compound) their hydrophobic moieties may enter the non-polar core of the bilayer. Modeling studies suggest that a specific molecular conformation may also be required for partitioning of, for example, verapamil into the bilayer (120).

Since substrates gain access to Pgp from within the membrane, the lipid–water partition coefficient, *K*_lip_, is an important molecular property affecting their interactions with the transporter. Lipid bilayers are multilayered, amphipathic structures (essentially highly ordered liquid crystals), and cannot be readily mimicked by isotropic systems such as octanol or olive oil. Experimental measurement of *K*_lip_ values using lipid bilayers is thus important for a quantitative understanding of the contribution of substrate partitioning to interaction with Pgp. *K*_lip_ values for Pgp substrates, whose structures are extremely diverse, range from 50 to 10^5^ (111, 112, 114, 122–126). The high lipid–water partition coefficients of a series of polyoxyethylene alkylether detergents resulted in their concentration in a palmitoyloleoyl-PC (POPC) bilayer (112). Calorimetric measurements indicated that lipid partitioning was an entropy-driven hydrophobic event, driven by release of water molecules from the detergent when it entered the bilayer.

The passive diffusion and spontaneous flip–flop of small molecules across membranes decline exponentially with an increase in lateral packing density (127), which in turn depends on lipid composition and temperature. Native plasma membranes with high lateral packing density, such as these found in endothelial cells of the blood brain barrier, may thus make it easier for Pgp to establish a drug concentration gradient. Phospholipid bilayers exist in a rigid gel phase below the phase transition (melting) temperature, *T*_m_, and a fluid liquid-crystalline phase above this temperature. These two phases are characterized by different packing densities, diffusion rates, fluidity, and lipid conformations. In an effort to understand the effects of membrane biophysical properties on partitioning of Pgp substrates, Clay and Sharom measured *K*_lip_ for three structurally unrelated Pgp substrates (LDS-751, H33342, and MK-571) in DMPC and palmitoylmyristoyl-PC (PMPC) bilayers over a temperature range that spanned *T*_m_ (122). Membrane partitioning of all three drugs was greatly favored in the fluid liquid-crystalline phase relative to the more rigid gel phase. The volume of the lipid bilayer increases by 4% when it melts (128), so drugs can fit more easily into the additional space present in the membrane in the liquid-crystalline phase, resulting in higher partitioning. The membrane partitioning of these three substrates was greatly affected by small changes in acyl chain length and lipid phase state, implying that such changes in membrane properties can readily modify Pgp’s ability to bind drugs within the bilayer and transport them to the aqueous phase. Lipid rafts are small cholesterol- and glycosphingolipid-rich microdomains which display altered lipid composition, including acyl chain length, and also exist in a phase with reduced fluidity (the liquid-ordered phase, l_o_), compared to the bulk membrane. Pgp appears to be located within rafts in some cell types (see below), and may move in and out of them in a dynamic fashion, thus potentially changing its access to drug substrates within the bilayer.

### ATP binding and hydrolysis

Although the NB domains of Pgp are envisaged as being located in the cytoplasmic compartment, they appear to be almost completely dependent on the presence of membrane lipids for their catalytic activity. Early work showed that a Pgp-β-galactosidase fusion protein required phospholipids for ATPase activity (129). Detergent delipidation of Pgp results in complete loss of ATPase activity, which is readily and rapidly reversible by addition of lipids (96, 98, 130), suggesting that the loss of activity does not involve denaturation of the protein.

The ability of bulk exogenous phospholipids to protect Pgp catalytic activity from thermal inactivation was tested by Sharom and co-workers, using partially purified Chinese hamster Pgp in CHAPS solution (96). Asolectin, a mixture of soybean phospholipids, provided complete protection of the ATPase activity at 0.25 mg/mL, whereas various PC species did not. PS was also able to maintain the ATPase activity, and various PE species, either alone or mixed with PC, actually stimulated activity. Restoration of ATPase activity following detergent delipidation with Triton X-100 and deoxycholate required a different set of phospholipids, likely reflecting the intimately associated annular lipids that Pgp prefers (96). In general, short, unsaturated fluid lipids were better able to restore activity than longer, saturated species. The ATPase activity of purified Chinese hamster Pgp in CHAPS solution was altered by incubation with various phospholipids, some of which stimulated activity, while others caused inhibition (50). The best stimulatory lipid, dipalmitoyl-PE, was a mixed activator, increasing *V*_max_ and decreasing the *K*_M_ for ATP. Exogenous lipids are probably able to exchange into the annular lipid region of Pgp in the presence of small amounts of detergent, thus modulating protein function. Mixed activation is a common observation with Pgp-drug substrates. The ability of phospholipids to modulate ATP hydrolysis could also be because they are actually transport substrates, as discussed previously in the context of the documented flippase activity of the transporter.

Urbatsch and Senior solubilized Chinese hamster Pgp using OG and attempted to purify it in the absence of exogenous lipids (86). This resulted in completely inactive protein that could not be restored by reconstitution. When Pgp was purified in the presence of three natural lipid mixtures, activity was maintained, and they were able to reconstitute it into proteoliposomes of these lipids (86). They noted different effects on the level of ATPase activity and its stimulation by drugs, depending on the host lipid. The level of drug stimulation, which may reflect coupling between drug binding and ATP hydrolysis, was also reported by other groups to be increased after reconstitution of Pgp (87, 131), as was high affinity drug binding (98).

To explore the effects of lipid melting on catalytic activity, purified Pgp was reconstituted into synthetic phospholipid bilayers with a defined melting temperature, composed of DMPC (*T*_m_ = ~23(C) and PMPC (*T*_m_ = ~28(C) (132). Both ATP binding and ATP hydrolysis were found to be modulated by the phase state of the membrane. The kinetic parameters and activation energy for ATP hydrolysis, as well as *K*_d_ for ATP binding, were significantly different above and below the bilayer melting temperature, and there was a sharp discontinuity at *T*_m_. The binding affinity for ATP was higher in the fluid liquid-crystalline phase, whereas the *K*_M_ for ATP hydrolysis was lower in the rigid gel phase. No changes in these parameters were observed at the same temperatures with Pgp in detergent solution. Thus, the conformation and/or folding of the NB domains may be affected by the fluidity and/or packing density of the membrane, which in turn affects nucleotide binding and hydrolysis. The opposite effects observed for *K*_d_ and *K*_M_ are not contradictory, since these parameters can only be considered equivalent for a simple Michaelis–Menten reaction scheme, and the catalytic cycle for Pgp is likely much more complex, with multiple steps.

Using drug-induced modulation of ATPase activity as a surrogate measure of drug binding, Seelig and co-workers were able to estimate the thermodynamic parameters for ATP hydrolysis (133). They showed that although the membrane lateral packing density only modulates the overall ATPase activity by up to two-fold, it substantially affected the thermodynamics of the transporter, which operated in an enthalpy-driven manner at low packing densities, but was driven by entropy at high densities. Partitioning of highly lipophilic Pgp substrates into the bilayer, and their accumulation to high concentrations (see above), may increase the fluidity and reduce the lateral packing density of the membrane. However, this appears to have only small effects on the thermodynamics of ATPase activity (133).

A FRET study showed that the NB domains of Pgp lie close to the membrane surface (134). In addition, when expressed in *E. coli*, the C-terminal NB domain was associated with the membrane fraction, indicating that it might interact preferentially with lipid surfaces (135). Taken together, this suggests that Pgp’s NB domains may be in physical contact with the membrane surface, much like peripheral proteins, explaining why membrane fluidity and packing density affect their function. The lipid requirement for catalytic activity may be interpreted in two ways. Either the lipids are needed for stabilization of the TM regions which in turn stabilize the NB domains, or the NB domains interact with the membrane directly and require phospholipids for their catalytic function and, possibly, their structural integrity.

Rodent and human Pgp appear to differ in terms of their stability and lipid requirements for activity. The human protein typically displays very low basal ATPase activity compared to the Chinese hamster protein, but shows much higher levels of drug-stimulated activity. Reconstitution into lipid is often necessary for observation of any ATPase activity at all [for example, see Ref. (136)]. The drug-stimulated activity of human Pgp also appears to be highly dependent on the presence of cholesterol (137). Kodan et al. carried out a systematic study of factors promoting the stability of human Pgp expressed in insect cells (10). They noted a remarkable stabilizing effect of cholesteryl hemisuccinate (CHS) on the stability of the protein’s ATPase activity after DDM solubilization; only 50% of the activity remained after 1 h at 25°C in the absence of CHS, whereas 50% activity was still present after 30 days at 4°C in the presence of 0.02% CHS. CHS has been noted to stabilize other human membrane proteins after DDM solubilization (138).

### Drug binding

Measurement of equilibrium drug binding to Pgp in plasma membrane vesicles from MDR cells has been carried out using radiolabeled substrates and modulators (139). This approach is technically difficult because the hydrophobic compounds give rise to high levels of non-specific background association with the membrane. Fluorescence spectroscopic methods using purified Pgp in detergent solution and reconstituted proteoliposomes have allowed quantitative estimates of *K*_d_ for binding of many substrates and modulators, without the need to separate free and Pgp-bound drugs [reviewed in Refs. (140, 141)]. The affinity of Pgp for binding a large group of compounds with diverse structures covers a range of ~10^4^, from 37 nM to160 μM.

Because of the difficulty of directly assessing drug binding, several groups have used drug or modulator stimulation of Pgp ATPase activity as a surrogate measure. Changing patterns of substrate-induced ATPase stimulation in the presence of different lipids suggests that either drug binding itself, or the coupling between drug binding and ATP hydrolysis, is altered by the membrane environment. Following Pgp purification and reconstitution into three natural lipid mixtures, Urbatsch and Senior noted different effects on the level of stimulation of ATPase activity by three substrates and one modulator, depending on the host lipid (86). Other groups also reported that after reconstitution of Pgp the level of drug stimulation of ATP hydrolysis (87, 131), and the affinity of drug binding (98) were increased.

**Figure 4:**
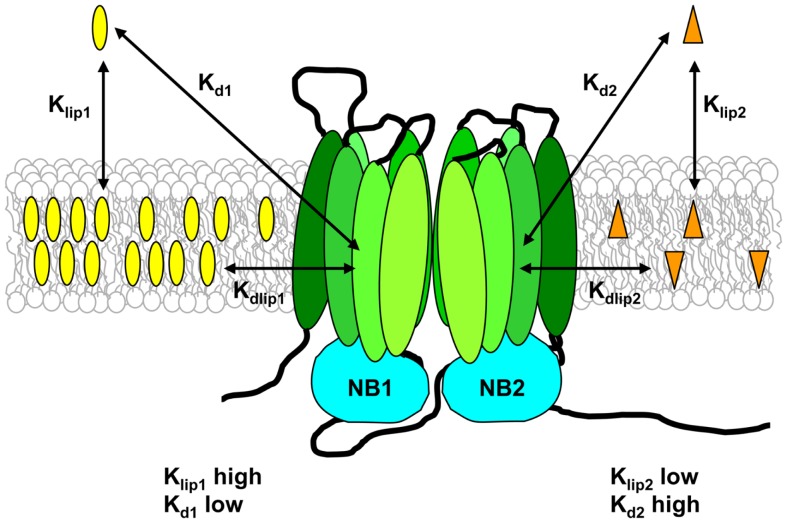
**Substrate–membrane relationships and their role in drug binding to Pgp**. Pgp substrates partition into the lipid bilayer from the aqueous phase in accordance with their lipid–water partition coefficients (*K*_lip_). The drug on the left of the figure has a high *K*_lip_ value and thus reaches a high concentration in the lipid phase, whereas the drug on the right of the figure has a low *K*_lip_ value, and therefore has a much lower membrane concentration. *K*_lip_ values measured for Pgp substrates are typically in the range 300–20,000. The apparent binding affinity (*K*_d_) is determined using aqueous drug concentrations, and depends on both drug partitioning into the lipid phase and drug binding to the transporter from within the membrane. The intrinsic affinity of Pgp for binding substrate from the lipid bilayer (*K*_dlip_) can be estimated from the values of *K*_lip_ and *K*_d_, and is relatively low, in the range 0.5–70 mM.

For a substrate to interact with Pgp, two distinct steps must take place. The drug must first partition from the aqueous phase into the bilayer, and then it must enter the binding pocket of the transporter. Seelig and co-workers proposed that Pgp functions optimally when the free energies for these two processes fall in a narrow range (114). The first step is defined by the lipid–water partition coefficient for the drug, *K*_lip_, and the second step is defined by the dissociation constant for binding of the drug to Pgp within the membrane, *K*_dlip_ (see Figure 4). The apparent binding affinity of Pgp for drugs measured from the aqueous phase, *K*_d_, is related to *K*_dlip_ by:

$$K_{\mathrm{dlip}}=\frac{K_{d}}{\left(\frac{V_{\mathrm{lip}}}{V}+\frac{1}{K_{\mathrm{lip}}}\right)}$$

where *V* is the total volume of the system, and *V*_lip_ is the volume of lipid (122). Thus experimental measurement of *K*_lip_ and *K*_d_ can provide an estimate of the “true” affinity of Pgp for binding drugs from within the lipid bilayer.

For reconstituted Pgp in three different lipid systems, a high-fluidity lipid (egg PC) gave a large increase in binding affinity (determined by fluorescence quenching) compared to a low-fluidity lipid (dipalmitoyl-PC; DPPC) for vinblastine and verapamil (6- to 15-fold reduction in *K*_d_ value), while daunorubicin showed a much smaller change (two-fold reduction) (124). *K*_d_ was also correlated with the value of *K*_lip_ for vinblastine and verapamil. The highest apparent binding affinity was observed for substrates that had the greatest partitioning into lipid, in other words, the highest concentration in the bilayer (Figure 4). This suggests that the bilayer drug concentration is a major determinant of binding affinity, in agreement with the vacuum cleaner model. Pgp may thus have a relatively low “true” affinity for binding within the bilayer (i.e., *K*_dlip_ is high), but the membrane highly concentrates its substrates. A large fraction of the free energy of drug binding to the protein from the aqueous phase was shown to be provided by the free energy of drug–lipid partitioning, on the order of 75% (122, 126). In concert with changes in binding affinity, different host lipids also produced an altered ATPase stimulation profile for verapamil. Higher drug-binding affinity led to higher levels of stimulation of activity, whereas the concentration of verapamil required for half-maximal stimulation remained unchanged (124).

The extent of partitioning of substrates into the membrane is thus important for their interaction with Pgp, because it essentially controls the effective concentration presented to the transporter. For example, the apparent binding affinity of *C. elegans* Pgp for paclitaxel and actinomycin D was increased 100- and 4,000-fold, respectively, when the protein was in a membrane environment compared to detergent solution (35). Seelig and co-workers were the first to address this idea directly, using the drug concentration required for half-maximal ATPase stimulation as an indirect measure of its binding affinity (142). By calculating the free energy of drug binding from the aqueous phase to Pgp in the membrane, and combining this with drug–lipid partitioning data, they were able to estimate the affinity of Pgp for binding three drugs from the lipid phase. *K*_dlip_ values were in the range 0.5–4 mM for three substrates (142), providing the first evidence that Pgp binds its substrates from the membrane with low affinity. Recent work by Clay and Sharom using reconstituted Pgp in DMPC and PMPC bilayers directly measured the *K*_d_ for binding to Pgp from the aqueous phase, and also the lipid–water partition coefficient for three structurally unrelated substrates (122). Estimated *K*_dlip_ values were high, in the range 9–70 mM, confirming that Pgp has an intrinsically weak interaction with substrates within the lipid bilayer.

Defined reconstituted systems provide a powerful tool to define the effect of bilayer physicochemical properties on drug binding. Romsicki and Sharom first made the interesting (and paradoxical) observation that the binding affinity for vinblastine, verapamil and daunorubicin was higher in gel phase DMPC bilayers, despite the fact that drug partitioning is substantially lower for gel phase lipid (124). More recent work using Pgp reconstituted into DMPC and PMPC bilayers showed that this relationship held true for the substrates H33342, LDS-751, and MK-571 (122); both *K*_d_ and *K*_dlip_ values are lower (i.e., binding affinity is higher) in rigid gel phase bilayers. Van’t Hoff plots of binding parameters over a wide temperature range showed different values of the binding free energy above and below the lipid phase transition. Since substrates must leave the lipid bilayer when they bind to Pgp, a less favorable drug–lipid interaction would encourage binding to the transporter. Analysis of the thermodynamic parameters for Pgp-drug binding in a manner which eliminated the contribution of the lipid was therefore carried out. Results indicated that both drug–lipid and drug–Pgp interactions contributed to the overall binding affinity, which was altered by both acyl chain length and lipid phase state, depending on the specific drug under consideration.

### Drug transport

Early work showed that small molecule fluidizers and surfactants could reduce Pgp transport function in plasma membrane vesicles and intact MDR cells, leading to increased accumulation of drugs and reversal of drug resistance (9, 84, 143–145). Transport inhibition does not appear to involve direct interaction between these agents and Pgp, and is probably linked to increases in membrane fluidity or permeability (146, 147).

A transport study using Pgp reconstituted into bilayers of the synthetic phospholipid DMPC found that ATP-driven transport of [^3^H]colchicine into reconstituted proteoliposomes determined by rapid filtration was more than two-fold higher at the *T*_m_ of 24°C than in the fluid liquid-crystalline phase at 32°C (8). Using Pgp in bilayers of DMPC and PMPC, the effect of bilayer phase state on the kinetics of ATP-driven transport was explored further (148). The initial rate of transport of the high affinity substrate tetramethylrosamine (TMR, a rhodamine derivative) was measured using a continuous real-time fluorescence assay over a time-scale of less than 1 min. Results showed that drug transport was modulated by the fluidity of the host membrane, with biphasic temperature dependence. The transport rate was high in rigid gel phase lipid, reached a maximum at the melting temperature of the bilayer, and then declined in fluid liquid-crystalline phase lipid. This behavior is unusual, as many membrane transport proteins show low or non-existent transport activity in rigid gel phase lipid, compared with activity in the fluid liquid-crystalline phase. Rigid gel phase lipid is poorly compressible, and it is thought that rate-limiting protein conformational changes are hindered in such an environment. It is possible that Pgp does not have a conformational barrier of this nature.

The rate of transport by Pgp likely depends directly on the drug concentration within the bilayer, and this, in turn, is controlled by the level of membrane partitioning of the substrate. Thus, the rate of drug transport may depend directly on the lipid–water partition coefficient, *K*_lip_. Rogers and Davis reported that partitioning of various *p*-alkylphenols into DMPC bilayers also showed biphasic temperature dependence (149). The value of *K*_lip_ showed a maximum at *T*_m_, declined substantially at temperatures higher than *T*_m_, and remained constant, or declined slightly at temperatures lower than *T*_m_, a pattern very similar to that seen for TMR transport by Pgp in bilayers of DMPC and PMPC (148). The temperature dependence of drug transport by Pgp may thus reflect the temperature dependence of *K*_lip_ for the substrate.

A recent study examined the effect of bilayer properties on Pgp-mediated transport of H33342 and LDS-751 in DMPC and PMPC bilayers, using real-time fluorescence measurements (122). Membrane partitioning data were also collected for these substrates, which allowed Pgp transport turnover numbers to be obtained in a reconstituted system for the first time. For both drugs, there is a clear discontinuity in transport rates above and below *T*_m_, however, in contrast to the previous studies using TMR, higher transport rates were observed in the liquid-crystalline phase relative to the gel phase. Different temperature dependence of partitioning of the three substrates into gel and liquid-crystalline lipid bilayers may be responsible for this difference in behavior. LDS-751 and H33342 displayed the opposite behavior to TMR; their lipid–water partition coefficients increased with temperature in liquid-crystalline phase bilayers.

Overall, these studies clearly indicate that the membrane phase state is an important factor affecting Pgp’s ability to transport drugs. One proposed strategy for reducing the transport activity of Pgp, thus reversing MDR, involves modifying the fluidity of the lipid environment (144). Since the effect of lipid fluidity on drug transport appears to vary depending on the specific substrate being transported, such an approach may not be feasible.

## Interactions of Pgp with Sterols

Cholesterol is a major component of mammalian plasma membranes, making up about 50% of the lipid content on a mole basis, and there have been many observations linking this sterol to Pgp function. Early work in intact cells suggesting the involvement of Pgp in cholesterol esterification was plagued by contradictions and confusion [reviewed in Ref. (123)] resulting from the use of Pgp modulators that later turned out to also inhibit cholesterol metabolism. Drug-selected MDR cells typically show changes in lipid composition, including cholesterol content, which may incidentally affect Pgp function. A study using cell lines where Pgp was inducible concluded that expression of the protein does not play a major role in cholesterol homeostasis (150), and that effects previously noted in drug-selected cells expressing Pgp might result from changes in other pathways that accompany selection. However, a possible physiological role for Pgp in intracellular cholesterol trafficking has been suggested (151). Using Pgp-deficient fibroblasts, replication of *Toxoplasma gondii* was shown to be critically dependent on Pgp, which played a role in the transport of host-derived cholesterol to the intracellular parasite.

Many studies investigating the role of cholesterol in Pgp function in intact cells and native membrane vesicles made use of methyl-β-cyclodextrin (MβCD), a cholesterol-extracting agent, to alter the amount of cholesterol in the plasma membrane (152–155). Essentially, all the cholesterol can be removed from membranes by this compound at a sufficiently high concentration, and inclusion complexes of MβCD with cholesterol can also be used to achieve controlled cholesterol repletion. However, MβCD was shown to directly inhibit the catalytic activity of Pgp, independent of its ability to extract cholesterol from membranes (123), calling into question the conclusions of studies that used this reagent.

Many groups have reported that the presence of cholesterol affects the catalytic activity of Pgp and its stimulation by substrates and modulators. When mouse Pgp was partially purified by hydroxyapatite FPLC using SDS, reconstitution into bilayers without cholesterol was reported to result in four- to five-fold reduced ATPase activity relative to bilayers where 20–40% cholesterol was incorporated (156). In addition, no stimulation of the activity by verapamil was observed unless cholesterol was included in the bilayer. Further studies showed that the lipids α-tocopherol and DPPC could also enhance verapamil stimulation, although they had no effect on basal ATPase activity (157). However, the ATPase activity of this Pgp preparation was very low, and it is possible that it was either partially denatured or not properly folded because of the somewhat harsh solubilization conditions, which likely strip away endogenous lipids. Callaghan and co-workers reported that the basal ATPase activity of partially purified hamster Pgp in PC–PE liposomes was increased ~two-fold on incorporation of 0–30% cholesterol (115). The fold-stimulation of ATPase activity induced by verapamil was reduced at higher cholesterol levels, although the absolute level of activity increased. Eckford and Sharom found that cholesterol had only a modest overall effect on catalytic activity. Purified Chinese hamster Pgp reconstituted into DMPC bilayers showed a small increase in basal ATPase activity, but a decrease in the level of verapamil-stimulated activity, with increasing cholesterol content from 0 to 30% (123). Cholesterol at high concentrations was able to partially restore the activity of delipidated Pgp, but less effectively than either PC or a PC-cholesterol mixture. Using purified reconstituted human Pgp, Kimura et al. reported that increasing cholesterol in the bilayer from 0 to 20% increased basal ATPase activity and modulated drug-stimulated activity (137).

However, cholesterol is not strictly required for Pgp function. Pgp purified using CHAPS is fully active (ATPase activity and drug transport) in proteoliposomes composed of synthetic phospholipids in the absence of added cholesterol [for example, see Refs. (122, 123, 132, 148)]. Perhaps this is because CHAPS-purified Pgp appears to retain an annular phospholipid layer around it (50). Alternatively, the sterol-like structure of CHAPS may interact with Pgp in a favorable manner, mimicking cholesterol. However, CHAPS does not stimulate Pgp ATPase activity like other detergents that are known to be substrates (96). It seems likely that the effects of cholesterol on both basal and drug-stimulated ATPase activity will differ depending on the specific detergent used to solubilize the protein, the extent to which endogenous lipids have been removed, and the lipid mixture used for reconstitution.

Cholesterol is well known to alter membrane packing, order, and fluidity. Such changes in the local environment may directly modulate the ability of Pgp to bind and hydrolyze ATP, and to transport drugs. Physicochemical changes in the membrane induced by cholesterol may also affect the concentration of drugs in the bilayer by changing their lipid–water partition coefficients. The inclusion of 30% cholesterol in DMPC proteoliposomes reduced Pgp’s binding affinity for ATP by ~2.5-fold (123). This is in agreement with a previous report that ATP-binding affinity decreased in bilayers with lower fluidity (132). Photoaffinity labeling of Pgp by the substrate [^3^H]azidopine was altered by yeast sterols, and cholesterol, suggesting that these lipids affected drug binding (158, 159). Later studies using fluorescence quenching to quantify drug interactions with purified reconstituted Pgp found that inclusion of cholesterol in the bilayer alters binding affinity (123, 124). The effects were dependent on the specific substrate used, with some drugs (e.g., vinblastine) showing a 10-fold increase in *K*_d_ value as cholesterol levels increased from 0 to 30%, while others (e.g., daunorubicin) showed essentially no change (123). These complex effects of cholesterol are different for individual drugs, and may be related to drug–lipid partitioning. Eckford and Sharom found that cholesterol had a large effect on drug partitioning into lipid bilayers, reducing the *K*_lip_ value by two- to nine-fold for several common Pgp substrates, with the exception of Hoechst 33342, which displayed a ~two-fold increase in *K*_lip_ (123). These differences may arise from the specific localization within the bilayer of drugs and cholesterol, which will modify the properties of different regions of the membrane selectively.

A water-soluble cholesterol analog, polyoxyethylcholesteryl sebacate, competed with daunorubicin for binding to Pgp (160), and cholesterol co-eluted with purified Pgp in detergent (137), however, it is still not clear whether these interactions are specific. Using the kinetics of drug-stimulated ATPase activity as a surrogate measure for drug-binding affinity, Kimura et al. reported that the presence of cholesterol in reconstituted proteoliposomes increased Pgp’s binding affinity for small molecular mass drugs (<500 Da), but not for larger drugs (800–900 Da) (137). They proposed that cholesterol may occupy the substrate-binding pocket simultaneously with smaller drug molecules, filling the empty space and thus promoting their binding (161). However, when drug binding to reconstituted Pgp was measured directly using fluorescence quenching, no relationship between drug size and the effect of cholesterol on binding affinity was found (123). A large drug (vinblastine) showed a large reduction in binding affinity at high bilayer cholesterol, whereas a small drug (daunorubicin) showed little change in affinity.

The effect of cholesterol on the ability of Pgp to flip fluorescent NBD–PC and transport three different substrates were explored in reconstituted proteoliposomes (123). A modest decrease in flippase activity was noted at 20–30% cholesterol, and the ability of vinblastine to compete for flippase activity was enhanced. Inclusion of cholesterol in the bilayer showed biphasic effects on the initial rate of transport of TMR and Hoechst 33342. Net transport into proteoliposomes is a balance between inward active pumping of substrate by Pgp and passive outward efflux through the bilayer. These results were explained by cholesterol altering the membrane permeability and partitioning of substrates, which affect transport indirectly (123).

Whether Pgp is directly involved in outward movement of cholesterol across the plasma membrane has been the subject of debate. The effect of Pgp on the transbilayer distribution of cholesterol in native membrane vesicles was monitored using cholesterol oxidation by cholesterol oxidase. (152). It was concluded that Pgp was responsible for ATP-dependent transport of cholesterol from the cytoplasmic to the extracellular leaflet of the membrane. However, the estimated rate of spontaneous cholesterol movement between membrane leaflets was very slow, with a half-time of ~10 min. This contradicts other reports of very fast intrinsic flip–flop [reviewed in Ref. (162)], which suggest that cholesterol does not require a transport protein to move between bilayer leaflets. Eckford and Sharom confirmed rapid flip–flop of two cholesterol analogs in native membrane vesicles and proteoliposomes, faster than the time-resolution of the cholesterol oxidase assay, and also failed to show Pgp-mediated flip–flop of cholesterol (123). Since Pgp mediates ATP-dependent translocation of SM (44), which has a high affinity for cholesterol (163), it may indirectly influence cholesterol distribution in membranes. It is unlikely that Pgp is directly involved in moving cholesterol between membrane leaflets.

## Lipid Rafts and Pgp

The cholesterol content of the plasma membrane is highly regulated and relatively invariant. However, some domains are selectively enriched in cholesterol, namely lipid rafts and caveolae, which are characterized by the presence of higher lipid order, lower density, and detergent resistance (164). In intact cells, dynamic movement of Pgp into and out of these more ordered, cholesterol-rich domains could potentially regulate its functions. Pgp has been proposed to localize in low density raft microdomains and caveolae in some cell and tissue types [for a detailed list, see Ref. (165)]. However, in one MDR cell line, Pgp was present in intermediate density Brij-96 domains that were biochemically and physically distinct from both classical low density lipid rafts and caveolae (166), and in another case, Pgp was found not to be associated with lipid rafts at all (167). Thus, any proposed role for these domains in functional modulation of Pgp must necessarily be cell type-specific. Typically, treatments that break up lipid rafts (e.g., MβCD) result in loss of Pgp from raft domains, and a decrease in its function [for example, see Ref. (168)]. However, interpretation of these results is complicated by the fact that MβCD has secondary effects besides cholesterol sequestration; it can extract other membrane components such as phospholipids (169), and it is also known to inhibit Pgp catalytic function directly (123). One other source of variation in assessing the association of Pgp with these domains is the methodology used to isolate lipid rafts. Several different detergents have been commonly used for their extraction from intact cells, including Triton X-100, Brij-96, and Lubrol, and detergent-free approaches using carbonate have also been employed [see Ref. (165)]. It has been suggested that lipid rafts domains are concentric layered structures, with different sensitivities to detergent extraction at their periphery compared to the central core (170). This may explain why Pgp is typically strongly associated with Lubrol- or Brij-96-based rafts, but less so with Triton X-100 rafts (171).

Perhaps not surprisingly, Callaghan and co-workers showed that Pgp was fully functional after reconstitution into liquid-ordered (l_o_ phase) membranes rich in sphingolipids and cholesterol, i.e., with a typical “lipid raft” composition (172). The main difference noted between Pgp behavior in raft-like proteoliposomes and in those composed of PC alone was that drugs were able to stimulate or inhibit ATPase activity at lower concentrations. This is compatible with reports that Pgp binds its substrates more tightly in less fluid lipid bilayers (122, 124).

## Detergents and Membrane Fluidizers as Pgp Modulators

Lipophilic compounds cross lipid bilayers by a three-step process involving partitioning into the interfacial region, diffusion through the hydrophobic core, and desorption from the opposite side of the membrane. Since many compounds, including Pgp substrates, are localized in specific regions of a lipid bilayer, the second step may be thought of as flip–flop (173), and is the slowest, rate-limiting step for movement of amphiphilic species across membranes. For example, doxorubicin flip–flop across lipid bilayers occurs with a half-time of ~1 min (174). The ability of substrates and modulators to interact with Pgp may depend on their ability to flip–flop between membrane leaflets. For example, some positively charged drugs and peptide modulators cannot interact with Pgp in intact cells if supplied on the extracellular side, probably because they have a very low rate of flip–flop to the inner leaflet (175). However, they can interact with Pgp in membrane vesicles, where the cytoplasmic leaflet is accessible. In support of this idea, substrate concentrations were found to be significantly lower in the cytosolic leaflet of intact cells expressing Pgp than in the cytosolic leaflet of inside-out membrane vesicles (176).

P-glycoprotein may handle classical modulators in exactly the same way as drugs, i.e., they are transported with hydrolysis of ATP (37, 177). The difference in the behavior of modulators and drugs may be related to their rate of flip–flop across the membrane (37). Pgp substrates were found to cross lipid bilayers relatively slowly (half-time for rhodamine 123 of ~3 min), while the transbilayer diffusion rates of several modulators were extremely fast. Pgp moves drugs and modulators (those that are transported) to the outer membrane leaflet, or they re-enter it after being moved to the extracellular medium. The rate of flip–flop to the inner leaflet is proposed to be slow for substrates, so that Pgp can keep pace, establish a drug concentration gradient across the membrane, and ultimately cause drug resistance. The long residence time of substrates in the inner leaflet also allows more opportunity for interaction with Pgp (174). For modulators, the rate of transbilayer flip–flop is so rapid that Pgp cannot keep pace or establish a concentration gradient, and MDR cells are not resistant to them. Work by Seelig and co-workers confirmed these ideas (176). Pgp in intact cells was found to transport substrates at a rate proportional to that of ATP hydrolysis, however, it could only prevent substrates from entering the cytosol if their rate of diffusion across the lipid bilayer was slow, in a range similar to that of Pgp-mediated efflux. This model suggests that for a modulator to be effective, it should bind to Pgp with high affinity and also have a high transbilayer diffusion rate (36, 177). If a compound modifies membrane properties to increase the rate of transbilayer movement of a drug sufficiently, it may be able to circumvent resistance without interacting specifically with Pgp. Thus, a second class of Pgp modulators may exist, consisting of agents such as surfactants and membrane fluidizers. In fact, several compounds that fall into this category are already known to reverse MDR (e.g., Pluronic block copolymers, Cremophor EL, Solutol HS15, Tween 80) (143, 178). Surfactants are ubiquitous in cleaning products, and are also present as additives in food and cosmetics. Since they are typically of very low toxicity, they may represent a useful class of Pgp modulators.

Evidence for the idea that Pgp-mediated MDR can be modulated by acceleration of passive drug permeation across the plasma membrane is contradictory. Tween 80 and Cremophor EL inhibited Pgp function in cell monolayers, increasing the apical-to-basolateral permeability and decreasing the basolateral-to-apical permeability of the substrate rhodamine 123 (179). These effects were related to the ability of the surfactants to increase membrane fluidity, in contrast with OG, which did not either modulate membrane fluidity or affect Pgp transport. However, polyoxyethylene surfactants that reversed MDR actually decreased lipid fluidity in plasma membrane vesicles from MDR cells, as assessed using several fluorescent probes (147), whereas surfactants that did not reverse MDR did not influence membrane fluidity. Low concentrations of Pluronic 61 were found to greatly increase the rates of phospholipid flip–flop and transbilayer movement of doxorubicin in lipid bilayers (180). Anesthetics (benzyl alcohol, chloroform, and diethylether) and non-ionic detergents (Tween-20, Nonidet P-40, and Triton X-100), increase membrane fluidity and the rate of transbilayer drug flip–flop (144). These compounds also abolish Pgp ATPase activity and drug binding, possibly by increasing membrane fluidity. Recent work in intact cells reported different results from experiments using liposomes and plasma membrane vesicles (146). Anesthetics were found to modulate MDR by accelerating transbilayer drug movement, whereas Pluronic P85, Tween-20, Triton X-100, and Cremophor EL had no effect on drug movement, and modulated MDR by inhibiting Pgp-mediated efflux. No correlation was found between the ability of surfactants to accelerate drug movement and their membrane fluidizing effects (146). Thus the molecular mechanism by which surfactants and membrane fluidizers inhibit the action of Pgp is still very much an open question.

## Conclusion and Future Prospects

P-glycoprotein is an unusual transporter, and various aspects of its function appear to be modulated by the lipid environment in novel and complex ways. Its mode of action as a hydrophobic vacuum cleaner and lipid/drug flippase make it especially sensitive to the properties of the surrounding lipid bilayer. Membrane composition, fluidity, and phase state all appear to be important parameters affecting Pgp stability, ATP binding, ATP hydrolysis, drug binding, and drug transport. Substrate interactions with the lipid bilayer play a critical role in the overall process of drug binding to Pgp, and are also modulated by the physicochemical properties of the membrane. It has been proposed that changing the properties of the host membrane may be a useful approach for clinical modulation of MDR. Clearly, an enhanced understanding of how all aspects of the Pgp catalytic cycle are affected by the local lipid microenvironment is essential if this strategy is to be successful. For example, the relationship between membrane partitioning and the binding affinity of Pgp substrates suggests one way to reduce or circumvent drug resistance. If a chemotherapeutic drug can be chemically modified to reduce its lipid–water partition coefficient, the ability of Pgp to transport it might be reduced. This would allow the drug to reach its intracellular targets, and thus increase its clinical effectiveness. Recent work on Pgp-membrane interactions has advanced to the point where it is now possible to measure thermodynamic and kinetic constants for the various steps of the catalytic cycle in model systems. Although the relationship between Pgp and its membrane environment is likely to be much more complex in living cells, this approach may provide a rational basis for novel strategies to overcome Pgp-mediated MDR in human tumors. It may also lead to a better understanding of the molecular mechanism of this enigmatic transporter.

## Conflict of Interest Statement

The author declares that the research was conducted in the absence of any commercial or financial relationships that could be construed as a potential conflict of interest.
